# ConvTransNet-S: A CNN-Transformer Hybrid Disease Recognition Model for Complex Field Environments

**DOI:** 10.3390/plants14152252

**Published:** 2025-07-22

**Authors:** Shangyun Jia, Guanping Wang, Hongling Li, Yan Liu, Linrong Shi, Sen Yang

**Affiliations:** College of Mechanical and Electrical Engineering, Gansu Agricultural University, Lanzhou 730070, China; jiasy@gsau.edu.cn (S.J.); wangguanping@gsau.edu.cn (G.W.); lihongling@gsau.edu.cn (H.L.); 12006@gsau.edu.cn (Y.L.); shilr@gsau.edu.cn (L.S.)

**Keywords:** plant disease identification, transformer, convolutional neural network, deep learning, ConvTransNet-S

## Abstract

To address the challenges of low recognition accuracy and substantial model complexity in crop disease identification models operating in complex field environments, this study proposed a novel hybrid model named ConvTransNet-S, which integrates Convolutional Neural Networks (CNNs) and transformers for crop disease identification tasks. Unlike existing hybrid approaches, ConvTransNet-S uniquely introduces three key innovations: First, a Local Perception Unit (LPU) and Lightweight Multi-Head Self-Attention (LMHSA) modules were introduced to synergistically enhance the extraction of fine-grained plant disease details and model global dependency relationships, respectively. Second, an Inverted Residual Feed-Forward Network (IRFFN) was employed to optimize the feature propagation path, thereby enhancing the model’s robustness against interferences such as lighting variations and leaf occlusions. This novel combination of a LPU, LMHSA, and an IRFFN achieves a dynamic equilibrium between local texture perception and global context modeling—effectively resolving the trade-offs inherent in standalone CNNs or transformers. Finally, through a phased architecture design, efficient fusion of multi-scale disease features is achieved, which enhances feature discriminability while reducing model complexity. The experimental results indicated that ConvTransNet-S achieved a recognition accuracy of 98.85% on the PlantVillage public dataset. This model operates with only 25.14 million parameters, a computational load of 3.762 GFLOPs, and an inference time of 7.56 ms. Testing on a self-built in-field complex scene dataset comprising 10,441 images revealed that ConvTransNet-S achieved an accuracy of 88.53%, which represents improvements of 14.22%, 2.75%, and 0.34% over EfficientNetV2, Vision Transformer, and Swin Transformer, respectively. Furthermore, the ConvTransNet-S model achieved up to 14.22% higher disease recognition accuracy under complex background conditions while reducing the parameter count by 46.8%. This confirms that its unique multi-scale feature mechanism can effectively distinguish disease from background features, providing a novel technical approach for disease diagnosis in complex agricultural scenarios and demonstrating significant application value for intelligent agricultural management.

## 1. Introduction

Plant diseases pose a significant challenge to agricultural production, leading to substantial losses in crop yield and quality [1]. Timely detection and accurate identification of these diseases empower farmers to implement effective prevention and control measures, thereby curbing disease spread. Moreover, such measures are essential for ensuring food security, minimizing the adverse environmental impacts of agricultural practices, and promoting sustainable agricultural development. Consequently, efficient and precise crop disease identification technologies have become indispensable tools in modern agriculture.

In recent years, deep learning has made remarkable advancements in crop disease recognition. Convolutional Neural Networks (CNNs), known for their exceptional feature extraction capabilities, have demonstrated outstanding performance across various conditions. CNN-based leaf disease identification methods have been widely applied to crops including apple, grape, tomato, cucumber, potato, rice, and wheat [2,3,4,5]. Kumar et al. [6] employed CNN models such as LeNet [7], VGG16 [8], ResNet50 [9], and Xception [10] for tomato leaf disease classification. These models were trained using 14,903 images from the PlantVillage dataset, covering nine disease categories and one healthy class. The results indicated that VGG16 achieved a detection accuracy of 99.25%. Additionally, Picon et al. [11] developed an adaptive deep residual neural network algorithm that effectively addressed multi-disease detection in practical scenarios, achieving 96% accuracy. As attention mechanisms gain traction, researchers have enhanced salient feature extraction by integrating these modules. For instance, JIANG et al. [12] added channel and spatial attention branches to ResNet18, achieving 98.25% accuracy for apple leaf diseases. Zhao et al. [13] combined a Spatial Attention Mechanism (SAM) with a Squeeze-and-Excitation Network (SE-Net) [14] to develop a parallel attention module, attaining 98.17% pest recognition accuracy. Transfer learning has also proven effective in mitigating limited data impacts. Rashid et al. [15] employed transfer learning with targeted architectural modifications to minimize detection errors. Their model, fine-tuned using a tomato leaf disease image dataset curated from PlantVillage, achieved an average training accuracy of 98.77% across all ten disease classes.

Despite CNNs’ success, performance plateaus as networks deepen. Beyond a critical depth, convergence slows and optimization challenges arise, with neither expanded datasets nor architectural changes significantly improving generalization [16]. Persistent challenges include: First, limited dataset diversity constrains model generalization [17,18]. Second, high inter-species morphological similarity, stochastic disease distribution, and complex symptom patterns—exacerbated by background interference (e.g., occlusions and lighting variations)—complicate feature extraction, leading to accuracy declines in field conditions [19]. Ahmad et al. [20] implemented four CNNs (VGG-16, VGG-19, ResNet-50, and Inception-V3) for tomato disease classification. Trained on both laboratory (simple background) and field-collected (complex background) datasets with feature extraction and fine-tuning strategies, Inception-V3 performed best, with laboratory accuracy 10–15% higher than field accuracy.

In recent years, transformer networks [21] have successfully addressed several limitations of traditional neural networks by introducing the self-attention mechanism, which offers a flexible, efficient, and scalable solution for processing long sequential data. The self-attention mechanism effectively captures long-range dependencies among regions in input images, enabling holistic understanding of plant disease characteristics. By stacking multiple transformer layers, models automatically learn and integrate multi-scale features, adapting to varying sizes and complexities of plant disease traits [22]. Zhang et al. [23] developed the vision transformer variant RCAA-Net, achieving 89% accuracy on the 2018 AI Challenge dataset (36,258 images, 61 diseases). Yu et al. [24] proposed an enhanced vision transformer module attaining 99.9% accuracy on PlantVillage. However, transformers exhibit deficiencies in capturing fine-grained local features compared to CNNs. For early-stage indicators such as minute spots or texture changes, their accuracy may be inferior [25]. Furthermore, with limited-scale datasets, transformers’ structural complexity and large parameter size render them prone to overfitting, diminishing their generalization capability in real-world applications and compromising performance on unseen disease images.

Current research predominantly focuses on single-model approaches, overlooking systematic integration of multiple architectures [26]. Combining complementary strengths could enhance the classification accuracy and robustness in agricultural applications. Comparative analysis demonstrates that merging CNNs with transformers substantially enhances feature representation [27]. Lee [28] proposed a Plant-CNN-Vision Transformer (Plant-CNN-ViT), integrating ViT, ResNet-50, DenseNet-201, and Xception. This hybrid achieved 100% accuracy on Flavia, Swedish, and Folio datasets and 99.83% on MalayaKew. Hu et al. [29] introduced FOTCA, employing an adaptive Fourier neural operator (AFNO)-based transformer for global features and convolutional downsampling for local features, demonstrating superior performance to conventional CNNs in accuracy, loss, inference speed, and convergence efficiency.

While existing methods have demonstrated remarkable progress in controlled settings, the accuracy and robustness of plant disease recognition systems continue to face significant challenges in complex natural environments [30,31]. Inspired by these findings, we propose ConvTransNet-S (Convolution-Transformer Network-S), a hybrid model capable of adapting to complex backgrounds, effectively extracting disease-specific features, and achieving accurate classification in real-world agricultural scenarios. The proposed model introduces a novel architectural framework that synergistically integrates complementary strengths of CNNs and transformers. Specifically, the framework leverages the transformer’s multi-head global self-attention mechanism to model long-range spatial dependencies across leaf surfaces, while retaining the CNN’s inductive biases through depthwise separable convolutions (kernel size = 3 × 3) for localized feature characterization. This design enables the hierarchical fusion of multi-scale visual patterns, enhancing the feature representation for subtle pathological variations. Furthermore, the model optimizes the relationship between multi-scale feature fusion and computational efficiency, thereby improving recognition performance. Experimental validation across multiple datasets demonstrates that, compared to existing CNN- and transformer-based models, ConvTransNet-S achieves superior accuracy with fewer parameters.

The primary contributions of this study are as follows:

1. We constructed a multi-crop dataset consisting of 10,441 field-condition images with complex backgrounds, encompassing 12 crop categories and 37 disease types, all of which were collected in real-world field environments.
2. We propose a hybrid network model named ConvTransNet-S, which achieves a dynamic equilibrium between local feature representations and global dependency modeling.
3. The model introduces the three key components: LPU, LMHSA, and IRFFN. This design effectively addresses the limitations of conventional CNNs in modeling long-range dependencies and mitigates the deficiencies of transformers in capturing local details, while significantly enhancing the stability of gradient propagation across network layers through hierarchical residual connections.

## 2. Materials and Methods

### 2.1. Experimental Data

**Figure 1:**
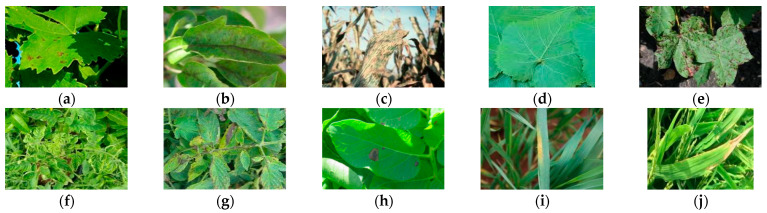
Exemplar images of plant disease in complex-background environments. (**a**) Grape leaf blight; (**b**) apple scab; (**c**) corn gray leaf spot; (**d**) grape healthy; (**e**) cotton bacterial blight; (**f**) tomato yellow virus; (**g**) tomato bacterial spot; (**h**) potato early blight; (**i**) wheat stripe rust; (**j**) rice bacterial blight.

To evaluate the model’s generalization capability, our validation framework necessitates not only laboratory-controlled single-background data but also the incorporation of field-collected samples under complex environmental conditions. The dataset utilized in this study consists of both a public dataset and a self-constructed dataset. The public dataset is sourced from the PlantVillage dataset, comprising 54,306 images spanning 14 plant species and 38 leaf categories, which include 26 types of diseased leaves and 12 types of healthy leaf images. The self-constructed dataset encompasses 33 disease leaf samples from 8 plant species (apples, corn, cotton, grapes, potatoes, rice, tomatoes, and wheat), along with healthy leaf samples from 6 of these species (excluding rice), resulting in a total of 39 distinct categories with 10,441 sample images, as illustrated in Figure 1. All images within this dataset were captured in natural agricultural settings, with their backgrounds exhibiting the following distinctive characteristics: (1) The presence of occlusions, such as foliage or crop stems, leads to partial information loss in the target objects. (2) The high similarity between targets and backgrounds in terms of color, luminance, and textural features results in indistinct boundary contours of the target objects. (3) The image backgrounds contain substantial non-target clutter (e.g., weeds and soil debris), generating complex visual noise that interferes with the target analysis. For data partitioning, this study employs a random split strategy, allocating the original dataset into training and test subsets at a 7:3 ratio. Specifically, the training set of the PlantVillage dataset contains 38,015 images, while its test set comprises 16,291 images. For the in-field dataset, the training and test subsets include 7309 and 3132 images, respectively. The partition details of the disease dataset under complex backgrounds are systematically summarized in Table 1.

### 2.2. Construction of Leaf Disease Identification Model

**Figure 2:**
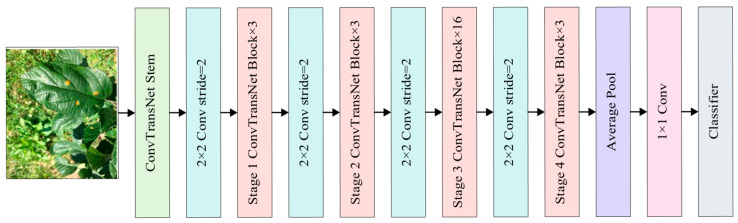
Overall architecture of ConvTransNet-S. More details are shown in Table 2.

The construction of the leaf disease identification model is depicted in Figure 2. The ConvTransNet-S model employs a stage-wise architecture design akin to CNNs, generating multi-scale feature maps through four hierarchical stages. At each feature extraction stage, the network utilizes a patch embedding layer, consisting of four convolutional layers interspersed with normalization layers, thereby achieving simultaneous feature abstraction and spatial downsampling. This hierarchical architecture enables a progressive downsampling mechanism for feature map resolution while systematically expanding channel dimensions to enhance the capacity for semantic representation. Within each stage, multiple ConvTransNet blocks are sequentially stacked to perform feature transformation while maintaining the input resolution. The network architecture varies the number of stacked ConvTransNet modules across stages: Stages 1, 2, and 4 each integrate three modules for efficient feature encoding, whereas Stage 3 incorporates 16 modules to enhance the hierarchical feature extraction capacity through dense contextual modeling. The model concludes with a global average pooling layer, a projection layer, and a 1000-way classification layer utilizing softmax [32].

**Table 2:** Architectures for ConvTransNet-S. The output size corresponds to the input resolution of 224×224. Convolutions and ConvTransNet blocks are shown in brackets with the number of stacked blocks (see also Figure 3, Figure 4, Figure 5 and Figure 6). $H_{i}$ and $k_{i}$ are the number of heads and reduction rates in LMHSA of stage i, respectively. $R_{i}$ denotes the expansion ratio in IRFFN of stage i.

Output Size	Layer Name	ConvTransNet-S
112×112	Stem	3×3, 32, stride 2 $\left[3\times 3,\;\;32\right]\times 2$
56×56	Patch Embedding	2×2, 64, stride 2
Stage 1	LPU LMHSA IRFFN	$\left[\begin{array}{c} 3\times 3,\;64\\ H_{1}=1,\;k_{1}=8\\ R_{1}=4 \end{array}\right]\times 3$
28×28	Patch Embedding	2×2, 128, stride 2
Stage 2	LPU LMHSA IRFFN	$\left[\begin{array}{c} 3\times 3,\;128\\ H_{2}=2,\;k_{2\;}=4\\ R_{2}=4 \end{array}\right]\times 3$
14×14	Patch Embedding	2×2, 256, stride 2
Stage 3	LPU LMHSA IRFFN	$\left[\begin{array}{c} 3\times 3,\;256\\ H_{3}=4,\;k_{3}=2\\ R_{3}=4 \end{array}\right]\times 16$
7×7	Patch Embedding	2×2, 512, stride 2
Stage 4	LPU LMHSA IRFFN	$\left[\begin{array}{c} 3\times 3,\;512\\ H_{4}=8,\;k_{4}=1\\ R_{4}=4 \end{array}\right]\times 3$
1×1	Projection	1×1, 1280
1×1	Classifier	Fully connected layer, 1000

#### 2.2.1. Stem Module

**Figure 3:**
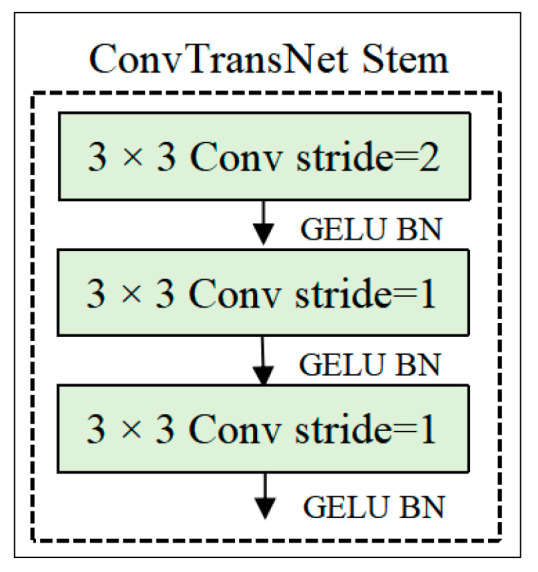
The structure of ConvTransNet Stem. The diagram illustrates the Stem module of ConvTransNet, which consists of three consecutive 3 × 3 convolutional layers. The first layer uses a stride of 2 for spatial reduction, followed by two layers with a stride of 1. Each layer is followed by a GELU activation function and Batch Normalization (BN).

The input image underwent fine-grained feature extraction via the Stem module, consisting of three stacked 3 × 3 convolutional layers. Notably, the initial convolutional layer employs a stride of 2 to produce 32 output channels, achieving spatial reduction while preserving essential local patterns. Two subsequent convolutional layers with stride 1 further enhance local feature representations. The resulting features are processed by multiple stacked ConvTransNet blocks for deep hierarchical learning. The ConvTransNet block architecture is systematically illustrated in Figure 3.

#### 2.2.2. ConvTransNet Block

**Figure 4:**
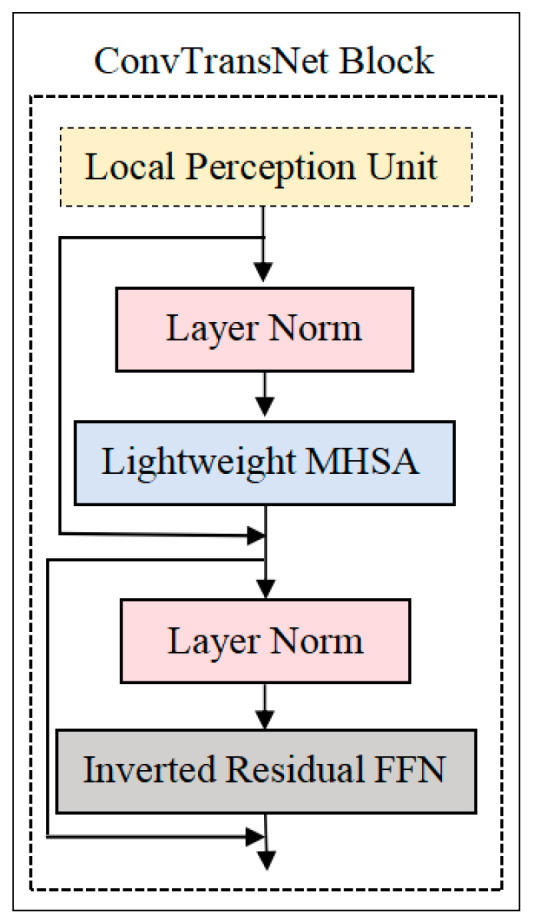
The structure of ConvTransNet block. The diagram illustrates the 3 core building blocks of ConvTransNet, which consist of three cascaded sub-modules: LPU, LMHSA, and IRFFN.

As depicted in Figure 4, the ConvTransNet module comprises three core components: the LPU for capturing fine-grained spatial dependencies, the LMHSA mechanism to model global contextual interactions, and an IRFFN designed for non-linear feature transformation with computational efficiency.

#### 2.2.3. Local Perception Unit

**Figure 5:**
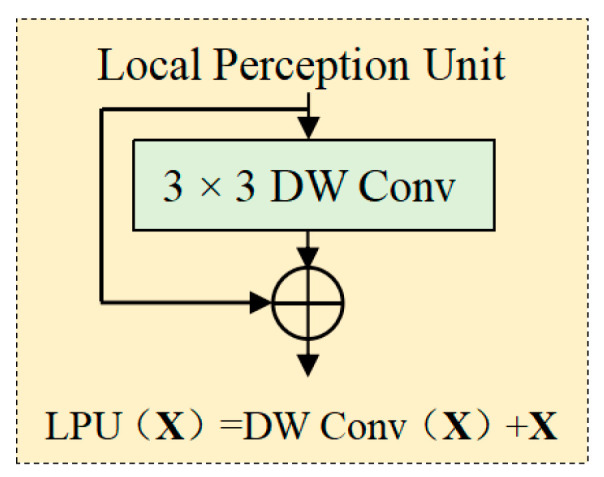
The structure of LPU. X is the input vector.

The proposed model addresses two critical limitations of standard transformers through the introduction of the LPU: (1) the disruption of translation invariance caused by absolute positional encodings and (2) the neglect of local relational and structural information within patches. The LPU is formally defined as follows:

(1) $$LPU\left(X\right)=DW\;Conv\left(X\right)+X$$

where $X\in R^{H\times W\times d}$ denotes the input tensor, H×W represents the spatial resolution (height × width) of the feature map at the current processing stage, and d indicates the channel dimension. DW Conv(X) corresponds to a depthwise convolution. The detailed architecture of the LPU is illustrated in Figure 5.

#### 2.2.4. Lightweight Multi-Head Self-Attention

To reduce the computational cost of the self-attention module, the LMHSA module is defined with h heads. A k×k depthwise separable convolution with a stride of k is utilized to decrease the spatial dimensions of K and V before the attention operation. Additionally, a relative position bias, B, is incorporated into each self-attention module. The corresponding lightweight attention is defined as follows:

(2) $$LightAttn\left(Q,K,V\right)=Softmax\;(\frac{{QK\prime }^{T}}{\sqrt{dk}}+B)V\prime$$

where $K\prime =DW\;Conv\left(K\right)\in R^{\frac{n}{k^{2}}\times d_{k}}$, $V\prime =DW\;Conv(V)\in R^{\frac{n}{k^{2}}\times d_{v}}$, $B\in R^{n\times \frac{n}{k^{2}}}$. The relative position bias matrix **B** is randomly initialized and learnable. The learned relative positional biases can be effortlessly transferred to matrices of different dimensions.

**Figure 6:**
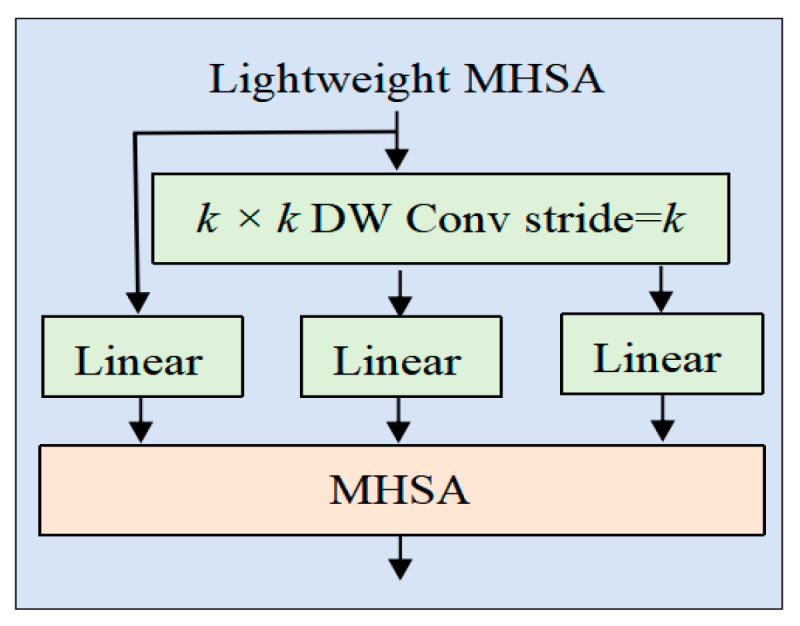
The structure of LMHSA. The LMHSA module performs spatial downsampling on the key (K) and value (V) tensors through a k×k depthwise separable convolution (with stride k).

$m_{1}\times m_{2}$ via bicubic interpolation: $B\prime \in R^{m1\times m2}$, B′=Bicubic(B). This design enables the proposed ConvTransNet to efficiently adapt to downstream vision tasks with minimal fine-tuning effort, as visualized in Figure 6.

#### 2.2.5. Inverted Residual Feed-Forward Network

**Figure 7:**
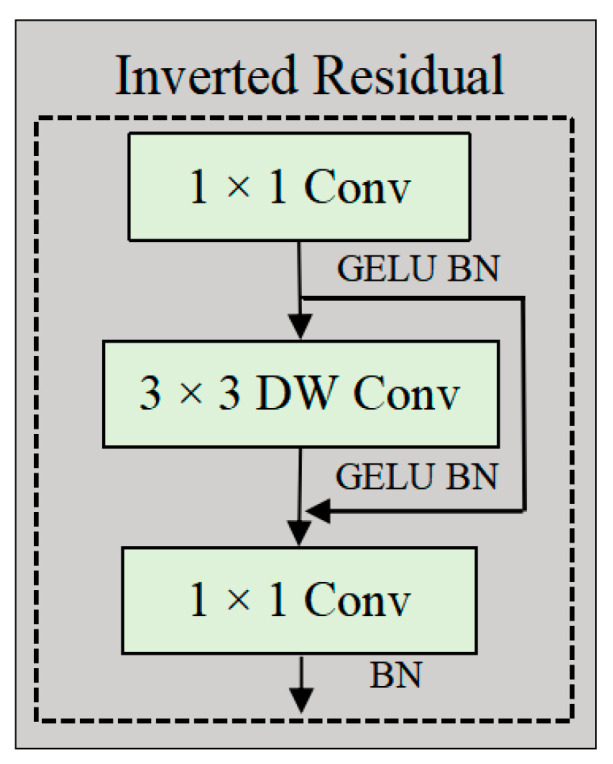
The structure of the IRFFN, including 1 × 1 convolution, GELU activation function, 3 × 3 depthwise separable convolution, and Batch Normalization (BN).

The IRFFN exhibits structural similarities with the standard Feed-Forward Network (FFN). It consists of an expansion layer, an activation function layer, a depthwise convolution layer, a projection layer, and a normalization layer. This enhanced module improves gradient propagation by optimizing residual connections through the reconfiguration of shortcut connections.

(3) $$FFN\left(X\right)=GELU\left({XW}_{1}+\;b_{1}\right){\;W}_{2}+b_{2}\;$$

where $W_{1}\in R^{d\times 4d}$ and $W_{2}\in R^{4d\times d}$ denote the weight matrices of the two linear layers, respectively. The terms b1 and b2 represent the corresponding bias terms. Figure 7 provides a schematic diagram of the IRFFN.

(4) $$IRFFN\left(X\right)=Conv(F(Conv(X)))$$

(5) $$F\left(X\right)=DWConv\left(X\right)+X$$

Through the combination of the aforementioned three components—LPU, LMHSA, and IRFFN—the ConvTransNet module can be expressed as:

(6) $$Y_{i}=LPU(X_{i-1})$$

(7) $$Z_{i}=LMHSA\left(LN\left(Y_{i}\right)\right)+Y_{i}$$

(8) $$X_{i}=IRFFN(LN(Z_{i}))+Z_{i}$$

where $Y_{i}$ and $Z_{i}$ denote the output features of the LPU and LMHSA modules in the *i*-th module, respectively. LN denotes Layer Normalization.

The detailed architecture hyper-parameters are shown in Table 2 [33].

### 2.3. Experimental Environment

All experiments in this study were conducted on a unified server platform with the following specifications: CPU: 12th Gen Intel Core i7-12700H Processor (Intel, Santa Clara, CA, USA); GPU: NVIDIA A10 with 9216 CUDA cores and 24 GB GDDR6 memory (NVIDIA, Santa Clara, CA, USA); core clock speed: 1563 MHz. The software stack utilized includes PyTorch 1.7.0, CUDA 10.0, and Python 3.6. Models were trained for 100 epochs.

### 2.4. Model Evaluation

To evaluate the performance of the model, we employed multiple metrics, including accuracy, inference speed, computational complexity (GFLOPs), and the number of parameters. Accuracy (abbreviated as Acc and measured as a percentage) is defined as the ratio of correctly classified samples to the total number of samples, calculated as follows:

(9) $$Acc=\frac{N_{Ture}}{N_{Sum}}\times 100\%$$

where NTrue is the number of correctly predicted test samples and Nsum is the total number of test samples.

## 3. Experimental Results and Analysis

### 3.1. Performance Comparison of Different Classification Models

To validate the efficacy of the ConvTransNet-S model, this study conducted a comprehensive comparative analysis with representative state-of-the-art recognition models spanning multiple architectural paradigms. The benchmark models include mainstream architectures: CNN-based models such as EfficientNetV2; transformer-based models, such as Vision Transformer (ViT) [34], and hybrid architectures, including Swin Transformer [35], which utilizes hierarchical windowed attention; ConFormer [36], which enhances self-attention through convolution; CrossViT [37], which employs multi-scale patch fusion; and MobileFormer [38], which enables bidirectional CNN–transformer interaction.

The classification performance of all benchmark models on the PlantVillage dataset is summarized in Table 3. Collectively, these models exhibit a strong performance, with the majority achieving recognition accuracy rates exceeding 90%. Among them, the EfficientNetV2 architecture stands out due to its low computational complexity in terms of the GFLOPs and parameter count, showcasing superior efficiency compared to other benchmarks. However, this reduced parameter capacity adversely affects the recognition accuracy and fails to yield significant improvements in inference speed. In our experiments, the EfficientNetV2 model achieved a recognition accuracy of 95.47% with an average inference time of 16.06 ms per image. This performance indicates that it does not attain an optimal balance between recognition precision and computational efficiency when compared to hybrid architectures. The transformer-based model achieved a recognition accuracy of 98.65% with an average inference time of 8.42 ms per image, highlighting the potential of transformer-based approaches to match or even surpass traditional CNNs in image classification tasks. However, achieving such high accuracy typically requires training on large-scale datasets, which not only increases the parameter count but also demands significant computational resources. Although ViT models demonstrate strong performance in ImageNet classification tasks, they tend to underperform relative to CNNs in fine-grained agricultural disease classification—even under similar model complexity. Notably, the ViT-Base architecture comprises 86.4 M parameters, requiring approximately 345.6 MB of memory for inference. Such a large parameter volume not only demands high-performance hardware but also imposes strict constraints on memory bandwidth and storage. For example, deploying ViT on agricultural drones or handheld scanners—devices typically equipped with low-power CPUs and limited RAM—would likely lead to sluggish inference speeds and potential memory overflow. The Swin Transformer reduces computational complexity and achieves linear computational complexity The Swin Transformer achieves linear computational complexity with respect to image resolution via hierarchical modeling and shifted window-based self-attention. However, the merging of neighboring patches during hierarchical feature aggregation may result in the loss of critical local fine-grained details necessary for agricultural pathology recognition. CrossViT enhances global contextual modeling by introducing a dual-branch cross-attention mechanism that establishes inter-scale dependencies between local fine-grained patches and global coarse-grained regions. This architecture demonstrates superior capability in capturing long-range spatial relationships compared to standard ViTs, which is particularly beneficial for agricultural pathology recognition that requires multi-scale disease analysis. While ConFormer improves the feature representation capacity through its dual-branch architecture (convolutional and self-attention pathways), the inherent computational overhead of its parallel processing mechanism results in significantly higher resource consumption. Notably, in our experiments, ConFormer achieved the lowest recognition accuracy (83.99%) among all the benchmark models, underperforming both pure CNNs and hybrid architectures. MobileFormer employs parallel branches and lightweight feature bridging modules to design efficient attention mechanisms for feature fusion. However, the model exhibits notable sensitivity to input image resolution, with inference latency increasing significantly at reduced resolutions. In our experiments, MobileFormer achieved an average processing time of 22.59 ms, revealing the inherent performance bottlenecks exposed by the demand for dynamic resolution adaptation in agricultural application scenarios.

As demonstrated in Table 4, the four variants of the ConvTransNet model exhibit exceptional performance in accuracy, inference time, GFLOPs, and parameter count, achieving significant enhancements in recognition precision while improving computational efficiency. The ConvTransNet-S achieves an accuracy of 98.85% with 3.762 GFLOPs and 25.14 million parameters; its inference time is 7.56 ms. Notably, compared to all CNN- and transformer-based baseline models, our approach achieves superior accuracy with significantly fewer parameters and lower computational costs, demonstrating efficacy for plant disease recognition.

### 3.2. Application of the ConvTransNet-S Model in Field Environments

This study evaluated the capability of the ConvTransNet-S model for plant leaf disease recognition in natural environments. We conducted tests using a custom-built dataset containing 39 categories of leaf images across 8 plant species. The experimental results showed a marked reduction in recognition accuracy across all models when deployed in natural environments, compared to their performance on the single-background PlantVillage dataset. As detailed in Table 5, the ConvTransNet-S achieved a recognition accuracy of 88.53% on the 3132-sample test set, outperforming both EfficientNetV2 (74.31%) and ConvTransNet-B (82.11%). Furthermore, it maintained an inference speed of 8.31 ms, demonstrating a 47.2% acceleration compared to the Swin Transformer’s 15.30 ms. The ConvTransNet-S exhibits enhanced robustness and real-time processing capabilities in complex field conditions, attributed to its hybrid architecture that integrates convolutional operations with attention mechanisms. While maintaining inference efficiency comparable to other models in its series (ConvTransNet-XS/ConvTransNet-T), it achieves a 6.42% higher accuracy than ConvTransNet-B. This validates the efficacy of parametric optimization in establishing an optimal balance between model compression and performance retention. Experimental validation confirms that ConvTransNet-S effectively captures both local textural patterns and global contextual correlations of leaf diseases under complex background interference. These findings provide a solid foundation for practical agricultural deployment, showcasing the model’s significant potential for real-world applications in challenging field conditions.

### 3.3. Ablation Studies

ConvTransNet-S establishes a novel hybrid architecture through the deep integration of ResNet50’s efficient feature processing capabilities with ViT-S’s strengths in global feature extraction, providing an innovative solution for plant leaf disease detection tasks. To systematically evaluate the model’s comprehensive performance, this study designed multi-dimensional ablation experiments. Table 6 presents a detailed comparative analysis of three model architectures (ResNet50, ViT-S, and ConvTransNet-S) across four critical metrics: accuracy, inference time, GFLOPs, and parameter count. The experimental results indicate that the ResNet50 model achieves a commendable accuracy of 86.08% while also demonstrating computational efficiency, characterized by a remarkably short inference time of 6.19 ms. However, the model exhibits progressive performance degradation with increasing network depth, highlighting inherent limitations in its hierarchical feature learning capacity. In contrast, the ViT-S architecture demonstrates superior global feature modeling capabilities through self-attention mechanisms. Nevertheless, its classification accuracy (85.33%) and inference time (8.56 ms) underperform convolutional baselines. This aligns with known limitations of pure transformer architectures in capturing multi-scale local features, particularly when processing heterogeneous leaf disease morphologies, which compromises feature representation robustness. The Convolution-Transformer Synergy Network (ConvTransNet-S) achieves architectural breakthroughs by preserving the computational efficiency of ResNet50 while innovatively integrating the global contextual awareness of ViT-S. This approach effectively mitigates the depth-induced gradient collapse endemic to conventional CNNs. Furthermore, its cross-scale hierarchical attention fusion mechanism addresses the limitations of multi-granularity feature extraction, inherent in vanilla transformers. This hybrid architecture ultimately achieves optimal performance with an accuracy of 88.53%, representing improvements of 2.45% and 3.2% over the baseline models ResNet50 and ViT-S, respectively. In terms of computational efficiency, although ConvTransNet-S has a relatively high GFLOPs value of 3.726, it maximizes computational effectiveness through a hierarchical feature processing mechanism and a dynamic computing resource allocation strategy. Architecturally, ConvTransNet-S implements depthwise separable convolutions in shallow encoder stages for localized pathological pattern extraction while deploying deformable attention mechanisms in deep transformer blocks to achieve cross-region semantic aggregation. This empirical validation substantiates that ConvTransNet-S achieves an equilibrium between computational complexity and model efficacy.

### 3.4. Fine-Grained Performance Evaluation on Sub-Class Datasets

This study elucidates the paradigm-shifting potential of hybrid architectures in agricultural vision systems: ConvTransNet-S not only synergistically combines the inductive biases of CNNs for local texture sensitivity with the global contextual reasoning of transformers but also demonstrates exceptional cross-hierarchical feature representations with self-supervised scale invariance. This provides a new technical pathway to address practical challenges such as lighting variations and occlusion interference in complex field environments, laying an important theoretical foundation for real-time disease recognition systems in smart agriculture scenarios.

**Figure 8:**
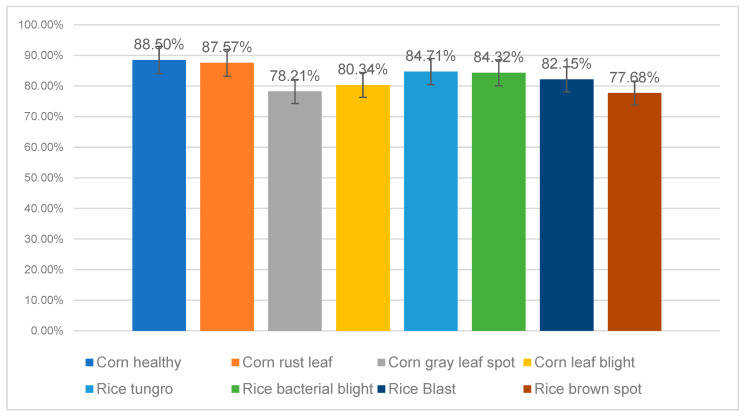
The recognition accuracy rate of diseased leaves across various categories. Tabular presentation of percentage values (top section) and a checklist of crop diseases (bottom section). The disease list includes options for corn (healthy, rust leaf, and gray leaf spot) and rice (tungro, bacterial blight, blast, brown spot, and leaf blight).

To gain deeper insight into the recognition capabilities of ConvTransNet-S for various plant diseases, we conducted a fine-grained performance evaluation utilizing subclass datasets, with particular emphasis on morphologically similar disease phenotypes across different infection stages. The test dataset comprised eight disease categories, four maize diseases (including healthy leaves) and four rice diseases, thereby forming a comprehensive evaluation framework. As illustrated in the classification accuracy results in Figure 8, the model achieved a peak accuracy rate of 88.50% in detecting healthy maize leaves, significantly outperforming its performance on diseased samples. This finding validates the model’s strong discriminative capacity in establishing decision boundaries for healthy versus diseased binary classification. The model exhibits robust recognition efficacy for disease categories with distinct morphological characteristics: corn rust achieved an accuracy of 87.57%, attributed to sensitive capture of chlorotic disease features; rice tungro attained 84.71%, benefiting from strong feature responses to chlorotic streaking patterns; and rice bacterial blight reached 84.32%, closely linked to precise localization of translucent stripe symptoms.

**Figure 9:**
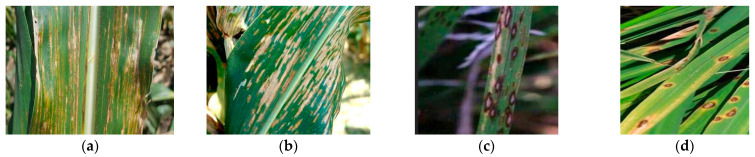
Visually similar diseases images. (**a**) Corn leaf blight; (**b**) corn gray leaf spot; (**c**) rice blast; (**d**) rice brown spot.

Nonetheless, we observed declining recognition accuracy for specific disease categories. For instance, accuracy rates for corn leaf blight and corn gray leaf spot were 80.34% and 78.21%, respectively. Through in-depth analysis of misclassified images, we identified two primary issues: First, the corn disease dataset is limited, causing uneven sample distribution. Second, both diseases exhibit irregular yellowish-brown streaks (5–15 mm) during mid-stage progression (14–21 days) with blurred lesion margins, increasing the misidentification risk. Despite larger sample sizes, significant confusion persists between rice blast (82.15%) and rice brown spot (77.68%), primarily due to shared characteristics: circular lesions with brown edges and grayish-white centers. Moreover, early infection stages show a morphological similarity exceeding discriminative thresholds. Figure 9 presents images of these visually similar diseases.

## 4. Discussion

The ConvTransNet-S model proposed in this study significantly enhances plant disease recognition performance in complex backgrounds by integrating the local feature extraction capabilities of CNNs with the global modeling strengths of transformers. Ablation experiments (Table 4) demonstrate that ConvTransNet-S achieves substantially higher accuracy on our self-constructed field dataset compared to ResNet50 and ViT-S. The core ConvTransNet block improves the capture of disease details through depthwise convolution in the LPU, incorporating the LMHSA downsampling strategy (Figure 5) with relative positional bias for modeling long-range dependencies. Additionally, the IRFFN optimizes gradient propagation paths (Figure 6). These innovations collectively enable robust feature extraction under field conditions, directly supporting deployment in agricultural edge devices. In terms of computational efficiency, ConvTransNet-S outperforms ViT (56.01 GFLOPs) and Swin Transformer (8.74 GFLOPs) with only 3.762 GFLOPs and 25.14M parameters (Table 3). This lightweight design (25.14M parameters) offers significant advantages for resource-constrained platforms such as agricultural drones or handheld scanners, where the model size directly impacts the real-time inference capability. During validation under complex backgrounds, the global average pooling strategy effectively mitigates background interference compared to ViT’s class token approach (Table 5). When combined with LMHSA’s downsampling, the model exhibits superior localization accuracy for indistinct diseases such as maize gray leaf spot. This precision in cluttered environments is critical for UAV-based crop monitoring systems requiring on-site disease diagnosis.

However, while demonstrating superior performance on both the PlantVillage dataset and our self-constructed in-field dataset, its robustness in broader agricultural scenarios warrants further validation. Key limitations include: (1) Environmental Variability: The performance was validated primarily under controlled field backgrounds, leaving its efficacy in extreme conditions untested. Seasonal changes in lighting or moisture may introduce false positives. (2) Crop and Disease Diversity: The current dataset covers 12 crops and 37 diseases, but the performance on rare diseases, newly emerging pathogens, or morphologically similar diseases across crops (e.g., rice blast vs. wheat stripe rust, requiring crop-specific fine-tuning) remains unexplored. (3) Edge Deployment Constraints: Although achieving a 7.56 ms inference time on an NVIDIA A10 GPU, the real-time performance on resource-limited devices (e.g., agricultural drones with embedded chips) requires further optimization via quantization, pruning, or knowledge distillation. (4) Data Imbalance and Annotation Costs: Recognition bottlenecks for diseases such as northern corn leaf blight (notably maize gray leaf spot with only 62 training images) stem from insufficient sample sizes and phenotypic similarities (Figure 9). Generative Adversarial Networks (GANs) could mitigate data scarcity but may introduce synthetic biases, while manual annotation of complex backgrounds remains labor-intensive—suggesting exploration of weakly supervised learning.

Future improvements will leverage strategies such as synthetic data augmentation [39], few-shot learning [40], and Grad-CAM-based interpretability analysis [41], alongside mobile deployment optimizations via knowledge distillation, dynamic inference, and hardware-aware NPU co-design. Comparative analysis shows that ConvTransNet-S maintains ViT-level classification accuracy with only 29% of ViT’s parameters, while its phased architecture (Figure 7) facilitates hierarchical multi-scale feature extraction—demonstrating marked superiority over ConFormer and MobileFormer in agricultural edge computing scenarios. In summary, ConvTransNet-S presents an efficient solution for agricultural disease identification. However, broader generalization across diverse environmental conditions, crops, diseases, and deployment platforms requires ongoing research.

## 5. Conclusions

To resolve the competing needs for local disease precision and global context awareness in field-based plant disease recognition, this study introduced the ConvTransNet-S hybrid architecture. This architecture establishes a multi-scale feature interaction system through the synergistic fusion of the local perception capabilities of Convolutional Neural Networks (CNNs) and the global modeling strengths of transformers. The core innovation of the model is embodied in its triple feature optimization mechanism: it enhances the ability to capture subtle textures of diseases while maintaining translation invariance through the Local Perception Unit (LPU), establishes long-range dependencies via the Long-Range Multi-Head Self-Attention (LMHSA), and optimizes gradient propagation while ensuring computational efficiency through the Improved Residual Feedforward Network (IRFFN). This approach resolves the fundamental trade-off in field-based plant disease recognition: achieving high-resolution local feature extraction and global dependency modeling under strict computational constraints, which traditional CNNs/transformers fail to jointly optimize. The core contribution of this paper can be summarized as follows:

1. On the PlantVillage dataset, ConvTransNet-S achieved an accuracy of 98.85% with only 25.14M parameters and 3.762 GFLOPs. This model outperforms EfficientNetV2, ViT and Swin-Transformer when evaluated under identical training protocols.
2. In robustness evaluations conducted under complex field conditions, ConvTransNet-S demonstrated significant superiority with a recognition accuracy of 88.53%, achieving improvements of 14.22% and 0.34% over EfficientNetV2 and Swin Transformer, respectively, highlighting its precision-separation capability for pathological features amidst cluttered backgrounds.
3. Ablation studies have confirmed the effectiveness of LPU in maintaining translation invariance and capturing subtle disease features. LMHSA reduces the computational overhead by 46.8% compared to the standard self-attention mechanism. Furthermore, the phased design strategy enhances hierarchical features without sacrificing model scalability. The findings demonstrate that ConvTransNet-S, by harmonizing local-global feature interactions and balancing accuracy with computational efficiency, provides a robust, efficient, and scalable solution for plant disease recognition in practical agricultural scenarios.

## Figures and Tables

**Table 1:** Composition of the complex-background plant disease dataset.

Type	Label Name	Number of Sample Images	Type	Label Name	Number of Sample Images
Apple	Apple healthy	516	Rice	Rice bacterial blight	1584
	Apple black rot	91		Rice blast	1440
	Apple rust	622		Rice brown spot	1595
	Apple scab	592		Rice tungro	1308
Corn	Corn gray leaf spot	62	Grape	Grape black rot	9
	Corn healthy	28		Grape esca (black measles)	11
	Corn leaf blight	178		Grape healthy	20
	Corn rust leaf	106		Grape leaf blight (isariopsis leaf spot)	12
Cotton	Cotton areolate mildew	30	Tomato	Tomato target spot	11
	Cotton bacterial blight	452		Tomato bacterial spot	76
	Cotton cercospora leaf spot	30			
	Cotton curl virus	337		Tomato early blight	78
	Cotton healthy	107		Tomato late blight	87
	Cotton target spot	68		Tomato mold leaf	76
	Cotton verticillium wilt	32		Tomato mosaic virus	49
Wheat	Wheat healthy	102		Tomato healthy	70
	Wheat septoria	97		Tomato septoria spot	119
	Wheat stripe rust	196		Tomato yellow virus	106
Potato	Potato healthy	49		Tomato spider mites Two-spotted spider mite	11
	Potato early blight	40			
	Potato late blight	44			

**Table 3:** Evaluation results of benchmark models on the PlantVillage dataset.

Model	Acc (%)	Inference Time (ms)	GFLOPs	Parameters (M)	Model Volume (MB)
EfficientNetV2	95.47	16.06	0.07859	0.121	29.58
Transformer	98.65	8.42	33.73	85.68	322.53
Vision Transformer	98.54	8.94	56.01	86.4	327.41
Swin Transformer	98.80	16.81	8.74	27.53	105.09
CrossViT	92.57	20.29	10.16	26.15	100.33
ConFormer	83.99	14.10	9.80	22.91	87.38
MobileFormer	96.91	22.59	329.89	6.46	24.63

**Table 4:** Evaluation results of ConvTransNet models on the PlantVillage dataset.

Model	Acc (%)	Inference Time (ms)	GFLOPs	Parameters (M)
ConvTransNet-B	94.49	22.47	8.689	45.72
ConvTransNet-T	89.79	10.05	0.596	9.49
ConvTransNet-XS	99.12	10.94	1.434	15.24
ConvTransNet-S	98.85	7.56	3.762	25.14

**Table 5:** Performance evaluation of comparative models on complex background dataset.

Model	Acc (%)	Inference Time (ms)
EfficientNetV2	74.31	16.18
Vision Transformer	85.78	8.17
Transformer	86.98	8.42
Swin Transformer	88.19	15.30
Con Former	86.73	23.50
CrossVit	86.88	12.49
Mobile Former	88.54	21.60
ConvTransNet-B	82.11	8.91
ConvTransNet-T	76.91	8.32
ConvTransNet-XS	88.06	8.21
ConvTransNet-S	88.53	8.31

**Table 6:** Ablation study on model component contributions.

Model	Acc (%)	Inference Time (ms)	GFLOPs	Parameters (M)
ResNet50	86.08	6.19	0.02359	89.97
ViT-S	85.33	8.56	3.52	22.02
ConvTransNet-S	88.53	8.31	3.762	25.14

## Data Availability

The original contributions presented in the study are included in the article, and further inquiries can be directed to the corresponding author.
